# Three-dimensional analysis of enamel surface alteration resulting from orthodontic clean-up –comparison of three different tools

**DOI:** 10.1186/s12903-015-0131-6

**Published:** 2015-11-18

**Authors:** Joanna Janiszewska-Olszowska, Katarzyna Tandecka, Tomasz Szatkiewicz, Piotr Stępień, Katarzyna Sporniak-Tutak, Katarzyna Grocholewicz

**Affiliations:** Department of General Dentistry Pomeranian Medical, University of Szczecin, Al. Powstancow Wlkp. 72, 70-111 Szczecin, Poland; Faculty of Mechanical Engineering Koszalin, University of Technology, ul. Raclawicka 15-17, 75-620 Koszalin, Poland; Department of Technology and Education, ul. Śniadeckich 2, 75-453 Koszalin, Poland; Clinic of Maxillofacial Surgery Pomeranian Medical, University of Szczecin, Al. Powstancow Wlkp. 72, 70-111 Szczecin, Poland

**Keywords:** Orthodontic clean-up, Orthodontic debonding, Residual adhesive removal, Enamel damage, Adhesive remnants

## Abstract

**Background:**

The present study aimed at 3D analysis of adhesive remnants and enamel loss following the debonding of orthodontic molar tubes and orthodontic clean-up to assess the effectiveness and safety of One-Step Finisher and Polisher and Adhesive Residue Remover in comparison to tungsten carbide bur.

**Materials and methods:**

Thirty human molars were bonded with chemical-cure orthodontic adhesive (Unite, 3M, USA), stored 24 h in 0.9 % saline solution, debonded and cleaned using three methods (Three groups of ten): tungsten carbide bur (Dentaurum, Pforzheim, Germany), one-step finisher and polisher (One gloss, Shofu Dental, Kyoto, Japan) and Adhesive Residue Remover (Dentaurum, Pforzheim, Germany). Direct 3D scanning in blue-light technology to the nearest 2 μm was performed before etching and after adhesive removal. Adhesive remnant height and volume as well as enamel loss depth and volume were calculated.

An index of effectiveness and safety was proposed and calculated for every tool; adhesive remnant volume and duplicated enamel lost volume were divided by a sum of multiplicands. Comparisons using parametric ANOVA or nonparametric ANOVA rank Kruskal-Wallis tests were used to compare between tools for adhesive remnant height and volume, enamel loss depth and volume as well as for the proposed index.

**Results:**

No statistically significant differences in the volume (*p* = 0.35) or mean height (*p* = 0.24) of adhesive remnants were found (ANOVA rank Kruskal-Wallis test) between the groups of teeth cleaned using different tools. Mean volume of enamel loss was 2.159 mm^3^ for tungsten carbide bur, 1.366 mm^3^ for Shofu One Gloss and 0.659 mm^3^ for Adhesive Residue Remover - (*F* = 2.816, *p* = 0.0078). A comparison of the proposed new index between tools revealed highly statistically significant differences (*p* = 0.0081), supporting the best value for Adhesive Residue Remover and the worst – for tungsten carbide bur.

**Conclusions:**

The evaluated tools were all characterized by similar effectiveness. The most destructive tool with regards to enamel was the tungsten carbide bur, and the least was Adhesive Residue Removal.

## Background

Orthodontic adhesive removal can be performed with different tools, including: hand instruments (scalers, pliers) and rotary instruments: sandpaper discs [1, 2], diamond burs [3], stainless steel burs [3], rubbers [4], tungsten carbide burs [1–6] and fiber-reinforced composite burs [7]. Kinetic removal of adhesive remnants by intraoral sandblasting has been described by Kim et al. [8] and ultrasonic clean-up - by Hosein et al. [9] as well as by Ireland et al. [10].

The most popular tools are tungsten carbide burs, which are rapid and more effective in relation to adhesive removal than Sof-Lex discs, ultrasonic tools, hand instruments, rubbers or composite burs. However, they remove a substantial layer of enamel and roughen its surface, thus should be followed by polishing [11].

No studies have been found that assess resin remnants or enamel loss following adhesive rest removal with a one step polisher and finisher or with Adhesive Residue Remover.

Iatrogenic enamel damage has been subjectively assessed under a scanning electron microscope (SEM) [1, 2, 12–16]. Numerous authors have used different indexes to rate enamel surface under SEM [17–23]. Enamel roughness after orthodontic clean-up has been measured using contact profilometry [24–26], a non-contact white-light 3D profilometry [27] or atomic force microscopy [7].

The first measurements of enamel loss were performed referring to the depth of a reference hole [28] or to a recessed steel marker [29]. Later, measurements were made using a profile projector [30], a null-point contact stylus system [31], Planer Surfometer [9, 10], laser scanning [32, 33] and 3D contact profilometry [34].

The aim of this study was to measure adhesive remnants and enamel loss after debonding orthodontic molar tubes and additionally to compare One-Step Finisher and Polisher, Adhesive Residue Remover and tungsten carbide bur referring to their effectiveness and safety.

## Methods

This study was found to be exempt from ethical approval (Ethical Committee of Pomeranian Medical University of Szczecin, Ref. No. KB-0012/09/01/2013). Informed verbal consent was obtained from all participants.

### Sample preparation

Thirty human third molars free from carious lesions, extracted for orthodontic reasons from patients aged 16–24 years were selected, based on the criterion of intact buccal surfaces free from cracks or restorations. They were stored in distilled water for 24 h before bonding, then cleaned using a low-speed bristle brush, rinsed for 10 s and dried with oil-free compressed air. For the purpose of 3D scanning, the experimental teeth were embedded in impression silicone (Bisico S1 Soft, Bisico, Germany) in order to prevent unnecessary movement during manipulation.

### Bonding, debonding and clean-up procedures

Following a 20 s etching with 35 % phosphoric acid (Ultra Etch, Ultradent, USA) molar tubes (ERA, Farfield, USA) were bonded directly, using chemical-cure orthodontic adhesive (Unite, 3M, USA), similarly to the clinical conditions: at the centre of each buccal surface, parallel to the long axis of the crown. The teeth with tubes bonded were then stored in 0.9 % saline solution for 24 h, rinsed with distilled water to prevent saline crystallization, dried with oil-free compressed air and debonded using ligature cutting pliers. The pliers were positioned similarly to in the clinical conditions, e.g. occlusally and gingivally in order to gently peel the tube from the enamel.

The clean up procedure was performed by the same operator under typical clinical conditions and continued until no macroscopically visible adhesive remnants could be found. Since macroscopic debonding patterns were different for individual molars, the authors decided not to assess the time needed to remove adhesive remnants. Three different tools were used for each set of ten specimens: a twelve-fluted tungsten carbide bur (123-603-00, Dentaurum, Pforzheim, Germany), a one-step finisher and polisher (inverted cone One gloss, Shofu Dental, Kyoto, Japan) and Adhesive Residue Remover (989-342-60, Dentaurum, Pforzheim, Germany).

### Assessment of adhesive remnants and enamel loss

All the specimens were scanned in blue-light technology, using a 3D optical scanner (Atos III, Triple Scan, GOM, Germany) before etching and after adhesive rest removal. Scanning was proceeded using a lens with a field of 170×130×130 mm, to the nearest 2 μm. The high scanner precision was maintained by a regular calibration procedure, as indicated by the manufacturer, thus an error study was not necessary. Two cameras observed the course of stripes projected on the teeth and point coordination was calculated for each pixel of the camera sensor. Scans of initial enamel surfaces were used as reference and those after adhesive removal – as virtual objects. Shape alteration of the enamel surface of each tooth was calculated using GOM Inspect software (GOM, Braunschweig, Germany). This procedure allowed the calculation of adhesive remnant height and enamel loss depth in every location of the buccal surface. Subsequently, the volume of adhesive remnant remaining and the volume of enamel lost were calculated for each tooth.

### Proposed index and statistical analysis

Data normality was tested using Shapiro-Wilk test at the level of significance α = 0.05. In order to compare variables between the groups of teeth, two tests were used at the level of significance of α = 0.05: a parametric ANOVA test for data of normal distribution and nonparametric ANOVA rank Kruskal-Wallis test - for data which did not show distribution normality.

The tools used should remove maximum residual adhesive with minimal enamel loss, thus an index of effectiveness and safety was calculated for every tool, according to the following equation:

$$ I=\frac{V_A+2{V}_E}{3}, $$

where I is a weighted average, V_A_ is adhesive rest volume and V_E_ is enamel loss volume; these are divided by a sum of multiplicands where enamel loss has been rated to be twice as harmful as adhesive remnants (enamel damage is irreversible and thus more detrimental).

Comparisons were made for adhesive remnant height and volume, for enamel loss depth and volume as well as for the proposed index of tool effectiveness and safety.

## Results

**Fig. 1:**
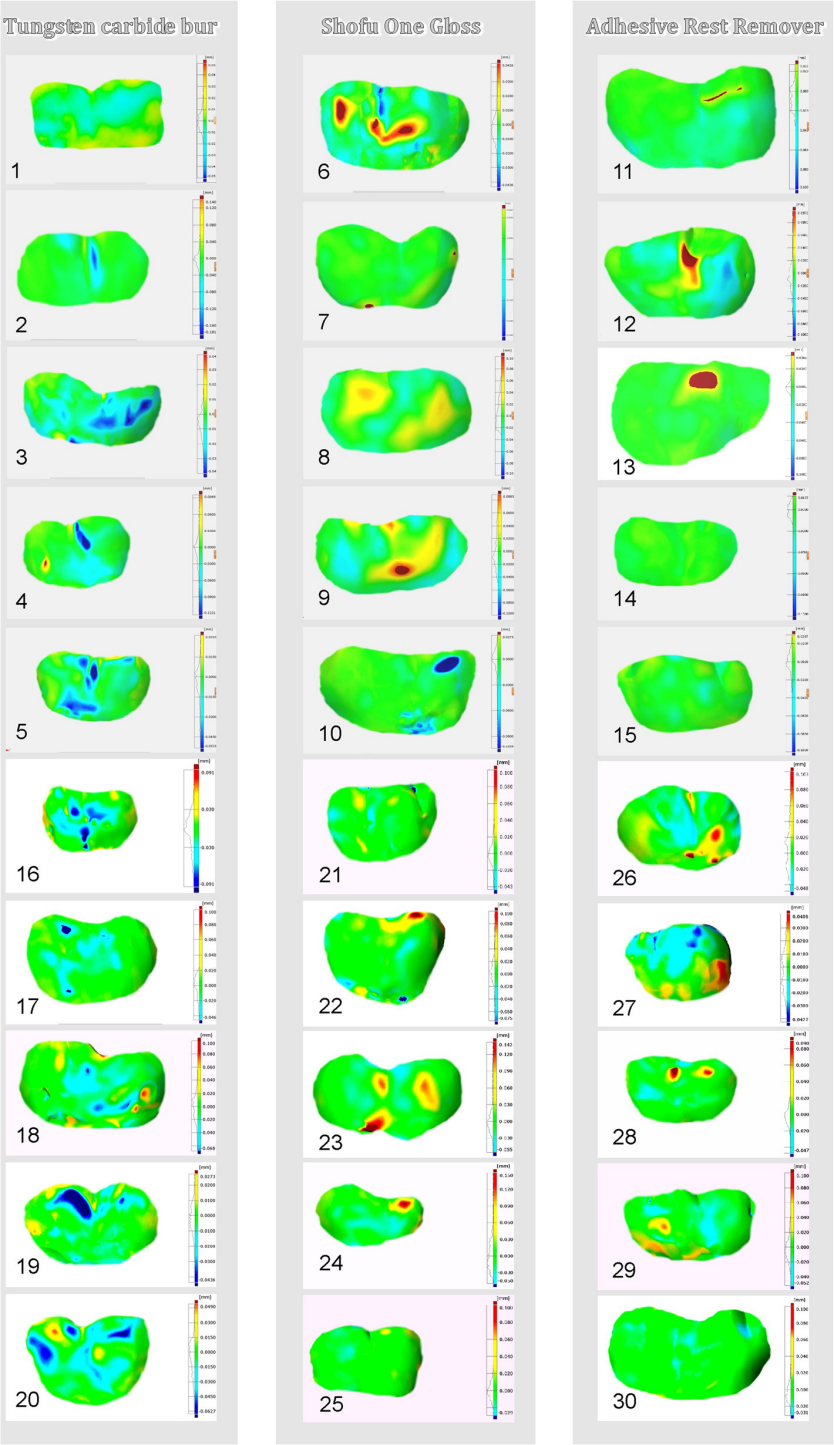
Superimpositions revealing shape alteration of the surfaces analysed

Superimpositions revealing shape alteration of the analysed surfaces are presented in Fig. 1.

The results concerning adhesive remnants height and volume after orthodontic clean-up using three different tools have been presented in Table 1.

**Table 1:** Adhesive remnants on particular teeth following adhesive removal

Tool	Adhesive remnants		
	Height [mm]		Volume [mm^3^]
	Mean (SD)	Min-Max	
Tungsten carbide bur	0.005838 (0.00471)	0.000008 – 0.028	0.079
	0.015758 (0.036838)	0.000126 – 0.3649	0.102
	0.004727 (0.003761)	0.000077 – 0.0179	0.0153
	0.01428 (0.019394)	0.000044 – 0.1104	0.065
	0.006079 (0.005262)	0.000001 – 0.031	0.032
	0.015872 (0.011378)	0.000007 – 0.069	0.302
	0.009011 (0.005749)	0.000009 – 0.031	0.1149
	0.14768 (0.014382)	0.000061 – 0.1489	0.3154
	0.006993 (0.005334)	0.000038 – 0.02726	0.0787
	0.010139 (0.008777)	0.000007 – 0.049011	0.07405
Shofu One Gloss	0.012092 (0.014155)	0.000002 – 0.0814	0.138
	0.0153979 (0.0143199)	0.000033 – 0.0744	0.074
	0.022308 (0.016684)	0.000058 – 0.0667	0.229
	0.0291329 (0.022995)	0.00004 – 0.1101	0.14
	0.00712 (0.005333)	0.000005 – 0.0275	0.084
	0.008655 (0.007351)	0.000007 – 0.040891	0.082445
	0.0185389 (0.020335)	0.000027 – 0.148891	0.26352
	0.03885 (0.046852)	0.000011 – 0.223663	0.3044
	0.026101 (0.02642)	0.000106 – 0.172313	0.232624
	0.00771 (0.006548)	0.000003 – 0.03969	0.12739
Adhesive Residue Remover	0.006229 (0.006331)	0.000004 – 0.0307	0.076
	0.024641 (0.038086)	0.000002 – 0.2111	0.281
	0.008671 (0.012583)	0.000001 – 0.0633	0.046
	0.00461 (0.003111)	0.000005 – 0.0127	0.05
	0.008 (0.005614)	0.00006 – 0.0297	0.087
	0.027002 (0.030753)	0.000019 – 0.247408	0.2213
	0.010685 (0.008191)	0.000001 – 0.041085	0.1413
	0.015896 (0.024687)	0.000005 – 0.178489	0.13467
	0.013959 (0.012721)	0.000022 – 0.068929	0.06608
	0.007048 (0.006245)	0.000033 – 0.039596	0.033121

No statistically significant differences in the volume (*p* = 0.35) or mean height (*p* = 0.24) of adhesive remnants were found (ANOVA rank Kruskal-Wallis test) between the groups of teeth cleaned using the various tools. Thus, the compared tools had a similar effectiveness. A superimposition of 3D scans made before bonding and those made after adhesive removal (Fig. 1.) suggests that adhesive remnants were left mainly in pits and fissures.

Enamel loss depth and volume resulting from orthodontic clean-up using three different tools have been presented in Table 2. Statistically significant differences (parametric ANOVA test) were found related to the volume of enamel loss (*F* = 2.816, *p* = 0.0078), while the mean depths of enamel loss did not differ significantly (*p* = 0077). The most destructive tool was tungsten carbide bur and the least was Adhesive Residue Remover.

**Table 2:** Enamel loss on particular teeth after adhesive removal

Tool	Enamel loss		
	Depth [mm]		Volume [mm^3^]
	Mean (SD)	Min - Max	
Tungsten carbide bur	0.005726 (0.003982)	0.000042 – 0.189	0.158
	0.0375016 (0.05145)	0.000012 – 0.3661	3.92
	0.00974 (0.008096)	0.000034 – 0.0631	1.486
	0.01076 (0.009623)	0.000002 – 0.0539	3.55
	0.01446 (0.014448)	0.000001 – 0.0923	1.25
	0.025638 (0.02466)	0.000011 – 0.1439	2.998
	0.0141 (0.012199)	0.000004 – 0.0727	1.755
	0.018288 (0.013947)	0.000006 – 0.0709	1.994
	0.011003 (0.012159)	0.000003 – 0.0767	2.8412
	0.018033 (0.014741)	0.000027 – 0.07849	1.63595
Shofu One Gloss	0.00838 (0.00704)	0.000009 – 0.0506	0.636
	0.013435 (0.01055)	0.000057 – 0.0635	0.473
	0.013494 (0.007662)	0.000079 – 0.0308	0.18
	0.017028 (0.011577)	0.000058 – 0.0484	0.303
	0.02347 (0.038124)	0.000011 – 0.2251	2.99
	0.009936 (0.011993)	0.000009 – 0.12439	3.577
	0.016158 (0.020371)	0.000014 – 0.20527	2.18648
	0.013648 (0.010161)	0.000003 – 0.0554	1.2456
	0.018005 (0.010717)	0.000033 – 0.0495	0.917376
	0.007047 (0.005541)	0.000002 – 0.02898	1.1427
Adehsive Residue Remover	0.012473 (0.007595)	0.000005 – 0.0351	0.5
	0.006229 (0.006331)	0.000004 – 0.0694	1.04
	0.00417 (0.0032)	0.000025 – 0.0175	0.109
	0.00546 (0.003323)	0.000013 – 0.0158	0.114
	0.007599 (0.006932)	0.000014 – 0.0375	0.388
	0.017349 (0.009953)	0.000009 – 0.04798	1.0587
	0.0134014 (0.008185)	0.000009 – 0.04272	0.5877
	0.008962 (0.007083)	0.000003 – 0.04717	1.53533
	0.012287 (0.009107)	0.000034 – 0.07611	0.60592
	0.009388 (0.005774)	0.00001 – 0.03067	0.646879

Comparison (parametric ANOVA test) of the proposed new index I between the tools revealed highly statistically significant differences (*p* = 0.0081), supporting the best value for Adhesive Residue Remover and the worst - for tungsten carbide bur.

## Discussion

Many studies evaluated adhesive remnants using Adhesive Remnant Index (ARI) [16, 17, 19, 21, 23, 35–40], however this surface assessment method does not allow the measurement of adhesive height or volume.

The present study investigated the cumulative effect of acid etching, debonding and adhesive removal. As the method of direct blue-light 3D scanning eliminates the reflections, the teeth can be scanned directly and there is no need for sputtering or making plaster models, resulting in higher accuracy. Most recently – this method was used to assess adhesive remnants and enamel loss after debonding molar tubes [41] and proved to be precise and reliable. In this study the authors used it to analyse the effect of grinding adhesive remnants.

This is the first study directly measuring adhesive remnants and enamel loss resulting from orthodontic clean-up. Moreover, this is the first study to assess the effect of adhesive removal with one step finisher and polisher as well as Adhesive Residue Remover.

Pont et al. [21] found no correlation between the amount of adhesive remnants and scoring of enamel surface after debonding and clean-up. Thus, no analysis of adhesive remnants before clean-up was presented in this study. However, adhesive remnant volume after debonding molar tubes has been presented elsewhere [41].

The amount of adhesive remnants after the clean up procedure depends on the operator, surface topography (fissures and porosity retain more adhesive) as well as on the tool used. It can be supposed that every tool may cut off the enamel especially in convex areas, causing enamel faceting, whereas remnant adhesive may be left in pits and fissures.

An elastic rotary instrument — a green rubber wheel has long been used for orthodontic adhesive removal by Gwinnet and Gorelick [4], who have concluded that it was the most effective (compared to green stone, white stone, sandpaper discs, tungsten carbide bur, steel bur or acrylic steel bur), giving a macroscopic polish; fine scratches were visible only microscopically and could easily be removed using pumice prophylaxis paste. It is interesting, that this tool, described as more efficient and less destructive than the most popular tungsten carbide burs has never been reported in any later studies.

It can be supposed that an elastic tool adapts its shape to the tooth surface, following the pits and fissures. Thus, prominent areas were ground less than by the tungsten carbide bur.

Rubber wheels may have different abrasive particles and different binders. One Gloss employs aluminium dioxide and silicone dioxide as an abrasive and the abrasive delivery medium is polyvinylsiloxane [42].

Adhesive Residue Remover is a stiff abrasive tool, with an appearance similar to a semi-transparent stone or rubber. No studies, which describe its use for orthodontic adhesive removal could be found. The first author contacted the manufacturer, asking for its composition, and was informed that this tool is made of epoxy resin and glass. We assume that epoxy resin is softer than enamel and while abraded, the abrasive particles of the tool are exposed. The detailed composition of the abrasive particles remains unknown, however it has been found that enamel loss is lower than for tungsten carbide bur or Shofu One Gloss.

It should be remembered, that a certain amount of adhesive is penetrating the etched enamel and this cannot be detected by surface scanning technique, constituting a possible limitation of this study.

Moreover, it should be noted, that enamel loss is one of the two aspects of iatrogenic enamel damage, e.g. removal of an enamel layer and enamel scratching (roughening).

## Conclusion

It can be concluded that the compared tools had a similar effectiveness. Referring to enamel loss, tungsten carbide bur had the most destructive effect, and Adhesive Residue Removal was found to be the safest.

## Abbreviations

3D: three-dimensional
ANOVA: analysis of variance
ARI: Adhesive Remnant Index
SEM: scanning electron microscope
